# Italian Teachers' Well-Being Within the High School Context: Evidence From a Large Scale Survey

**DOI:** 10.3389/fpsyg.2019.01926

**Published:** 2019-08-21

**Authors:** Barbara Barbieri, Isabella Sulis, Mariano Porcu, Michael D. Toland

**Affiliations:** ^1^Department of Social and Political Sciences, University of Cagliari, Cagliari, Italy; ^2^Department of Educational, School and Counseling Psychology, University of Kentucky, Lexington, KY, United States

**Keywords:** well-being, job satisfaction, teachers, PISA, measurement models, multilevel

## Abstract

This paper aims to investigate the relationship between Italian teachers' well-being, socio-demographic characteristics and professional background. Using data from the 2015 wave of the Program for International Student Assessment (PISA) we considered information collected by the questionnaire completed by a total of 6,491 teachers in the sampled schools. Moving from existing literature on teachers' well-being, we investigate several aspects related to the teachers' working environment, career motivation and investment, and job satisfaction. We assess the variability in the observed outcomes attributable to school factors and heterogeneity between disciplines. Measurement models are combined in a multilevel setting in order to define teachers' well-being on a broad perspective while accounting for the multiple sources of heterogeneity due to several factors (e.g., discipline, teacher professional background, and individual differences) occurring at different levels of the data structure. In general, results show that the teachers' positive perception of the working environment in terms of availability of adequate human and physical resources, and professional development opportunities, provide a substantial state of well-being at work, and are related to teachers' job satisfaction. Moreover, results highlight the key role of transformational leadership in defining a teacher's well-being. Findings and implications are discussed.

## Introduction

Research in different cultures indicate that school teachers are among those professionals with the highest levels of job stress (Stoeber and Rennert, 2008; Fiorilli et al., 2015a), often attributed to an excessive workload, poor relationships with colleagues, lack of suitable resources, constant changes within the profession, and inadequate salary (Santavirta et al., 2007; Simbula et al., 2012; Falco et al., 2013).

Italian teachers are in the last positions for the perceived social recognition of the profession (OCDE, 2014). Furthermore, in 2015 the Italian National Observatory on Health and Well-being of Teachers (ONSBI—Osservatorio Nazionale Salute e Benessere degli Insegnanti) research study highlighted the low satisfaction of the category for the physical (Guglielmi et al., 2012) and organizational conditions of schools (Fiorilli et al., 2015b). Although the OBSBI study was able to assess the risk levels of teachers' physical and psychological health and the factors that influence them, it was not very informative with respect to the factors that contribute to generating a condition of well-being among teachers. Studies on the school environment, and in particular on the teachers' role, have been focused primarily on the nature of the stress process, assuming that the context itself is overly demanding and characterized by lack of support toward teachers (Kyriacou, 2001). A more recent line of studies on teacher well-being at work, conversely, analyzes the working environment from a resource perspective, emphasizing how work environment and job characteristics explain motivation or work engagement despite high job demands (Bakker et al., 2007; Magnano et al., 2014). Moreover, literature highlights how job resources may be located at the level of the organization at large (e.g., career opportunities, job security), interpersonal and social relations (e.g., supervisor and co-worker support, team climate), or organization of work (e.g., participation in decision making; Bakker et al., 2007).

Thus, the central aim of the present study is to investigate, in a sample of 6,491 Italian high school teachers, how some professional and organizational factors (i.e., professional choice; professional development; principal leadership and colleagues support; perception of rich vs. poor work environment; management practices and polices) could be perceived by teachers as potential resources of well-being in terms of job satisfaction at work and satisfaction with respect to their profession. Understandably, a teacher's well-being at school is not only related to the professional context, but a teacher's personality or personal background can influence the way in which a teacher deals with factors within the school and with the demands of the profession. Consequently, we not only measure the individual well-being of teachers, but assess the school's contribution to overall teachers' well-being.

## Theoretical Framework

As it is well-known in literature, teaching is a highly demanding occupation (Travers and Cooper, 1996; Guglielmi and Tatrow, 1998; Kyriacou, 2001; Zurlo et al., 2007). Research suggests that the main stressors among teachers may include, low salaries, disagreement with colleagues or conflict with students' parents, redefinition of job conditions as a consequence of school reform, workload, role conflict and ambiguity, and poor working conditions (Travers and Cooper, 1996; Kyriacou, 2001; Zurlo et al., 2007; Benevene and Fiorilli, 2015; Guidetti et al., 2015; Falco et al., 2017; Girardi et al., 2018). Although many studies indicate antecedents of discomfort, stress and burnout, it's not until more recently that studies have observed the link between organizational health and teacher well-being (Hakanen et al., 2006; Skaalvik and Skaalvik, 2011; Miglioretti et al., 2013). Researchers have also shown how teacher well-being could be an important determinant of student learning outcomes and students' well-being, highlighting the importance to establish the determinants not only for teachers' negative outcomes, but also positive ones that are associated to their work (Denny et al., 2011; Converso et al., 2014; Guidetti et al., 2015). Form this perspective, the present study aims to investigate teachers' well-being within the theoretical framework of the Job Demand –Resource model (JD-R model), in which job resources are all those factors that can help employees in reducing job demands and related costs (Bakker and Demerouti, 2007; Barbieri et al., 2016; Farnese et al., 2017; Falco et al., 2018; De Carlo et al., 2019). In fact, job resources include factors capable of fostering personal growth, learning and development by providing feedback, enhancing task significance, and stimulating employees' motivation (Bakker et al., 2005; Bakker and Demerouti, 2007, 2014).

In light of the above, our goal in this study was to assess the school's contribution to teachers' well-being. More specifically, some professional and organizational dimensions have been considered as distinctive resources of the job, able to positively influence teachers' well-being. To this end, we considered professional choice (i.e., did the teacher actually want to be a teacher), and professional development as characteristics of job resources at the professional level; while the perception of rich vs. poor work environment (i.e., shortage of teaching resources, shortage of educational resources), practices and policies management (i.e., to take part in professional development activities) and principal leadership and colleagues cooperation as characteristics of job resources at the organizational level which are assumed to be related to teachers' well-being. We also considered teacher demographic factors as they relate to well-being [i.e., gender, work experience (i.e., years working as a teacher), and the type of subject taught (i.e., Humanities, Math-Sciences, Other)].

### Professional Choice

In educational literature it is recognized that teacher identity is a key influencing factor on teachers' sense of purpose, self-efficacy, motivation, commitment, job satisfaction and effectiveness (Day et al., 2006; Avanzi et al., 2012; De Stasio et al., 2017). A strong identification with social self (i.e., being a teacher), produces a positive effect on relationships and perception of effectiveness of their own role. Moreover, a perception of effectiveness of the own role is positively related to commitment to and a passion for teaching (Day, 2004) and to stay involved in school activities. According to self-determination theory (Deci and Ryan, 1985), people who carry out a chosen profession are more intrinsically motivated to engage in the activities of their job. Ryan and Deci (2000) also have described how extrinsically motivated activities can be internalized if people value the outcomes of these efforts and if people have access to supportive contextual conditions. In this study we considered the professional choice as a personal resource. An important extension of the JD-R model includes personal resources, as a further protective factor that can trigger positive outcomes (Bakker and Demerouti, 2014; Bakker et al., 2014). Some scholars (Xanthopoulou et al., 2007) observed that job resources influenced personal resources—conceived as the individuals' sense of ability to successfully control their environment—and this, in turn, had a positive influence on burnout reduction (Bakker et al., 2014). Following this observation, we considered the personal choice of being a teacher as a personal resource that is useful to understand and control the work environment. Ultimately, this resource can be strengthened and enhanced through organizational strategies (e.g., school's practices and policies oriented to a more participatory and inclusive processes management could have a positive influence on the teachers' sense of ability to successfully manage the school environment).

### Professional Development

Teacher satisfaction at work is related to the ways in which school policies encourage or facilitate career aspirations and development (Butt and Retallick, 2002). Several researchers have highlighted that professional development paths can have positive influences on outcomes such as teachers' satisfaction (Ullah and Jundran, 2014), attitude change, commitment to innovation (Desimone, 2009), and self-efficacy (Tzivinikou, 2015). Professional development can either be hindered by the school's organizational context or on the contrary, can contribute toward commitment to learning goals and collaboration in school (King, 2002). Different research on employees in different types of working environments have shown that personal development opportunity (Liu and Wang, 2001), training (Long et al., 2002), and opportunity for learning (Ng et al., 2006) explain employees' commitment to their organizations and occupational well-being (Weng et al., 2010; Barbieri et al., 2018).

### Work Environment

Researchers in positive psychology have identified the critical role that satisfying work plays in psychological well-being across various domains of human functioning (Lent, 2004; Doest et al., 2006). Numerous studies (see Hoy and Miskel, 1982; Huberman and Vandenberghe, 1999; Hallinger, 2003) have also underlined how workplace-related factors can positively influence job satisfaction and well-being. Such as content of the job, physical and material working conditions, school management, school climate, and interpersonal relationships (e.g., Hakanen et al., 2006). Ultimately, the intensity and frequency with which certain conditions are realized or missing will have consequences for the employee's well-being.

### Management Practices and Policies

The organizational domain of the teacher functioning at school has a markable influence on the teacher as an employee (Cherniss, 1993). For instance, the extent to which teachers are involved in school-based decisions determines the scope in which they sense control over expressing their values through their work (Friedman, 2003). Moreover, several researchers have emphasized that an important element of teachers' identities is related to their experiences in the school (Galloway et al., 1982; Mortimore et al., 1988; Woods et al., 1997). The culture of the school, its internal dynamics and organization, enable or hamper teacher “satisfaction,” “commitment,” and “motivation,” and ultimately the culture of the school has an impact upon a teacher's construction of their own teacher identity.

### Principal Transformational Leadership and Colleagues Cooperation

An important resource for teachers, as for most helping service professions, is to recognize and utilize a social support system to deal with work stress, prevent burnout (Kyriacou, 2001), and increase job satisfaction (Brough and Pears, 2004). Also, theoretical arguments suggest that transformational leadership will be positively associated with perceiving work as meaningful (Arnold et al., 2007; Benevene et al., 2018; Magnano et al., 2019). For instance, Piccolo and Colquitt (2006) have demonstrated a positive link between transformational leadership and employee perceptions of meaning in terms of these job characteristics. Some researchers have argued that this kind of leadership “gives meaningfulness to work by infusing work […] with moral purpose and commitment” (Shamir et al., 1993, p. 578). Also, the individual respect and support that a transformational leader exhibits for each employee should also apply to the actual work in which each employee is engaged. Transformational leaders inspire employees to transcend self interest and perceptions of their own limitations to become more effective in pursuing collective goals (Bass et al., 2003). They support employees in working toward the goals, such as by acting as a role model, stimulating them to engage in analysis, showing concern for them as individuals, and encouraging teamwork (Podsakoff et al., 1990). Studies have consistently shown that in the general population social support from a leader/manager and colleagues is positively associated with work engagement and well-being (Schaufeli and Bakker, 2004). In addition, researchers have shown the importance of the support provided by a leader not only in generating a sense of meaningfulness (Kahn, 1990), resilience, security, and general motivation (Ryan and Deci, 2001), but also in enhancing an intrinsic or extrinsic motivational role (Bakker and Demerouti, 2007). Laschinger et al. (2004) found that if a leader provides a more supportive environment for their employees, employees will adopt better work attitudes. Also, leaders who show appreciation and support may aid the worker in coping with the job demands, facilitate performance, and act as a protector against ill health (Väänänen et al., 2003). For instance, a study among Finnish teachers (Bakker et al., 2007) showed how leader/supervisor support maintains a high work engagement over time among employees. Other studies have shown that a high quality relationship with colleagues may alleviate the influence of job demands (e.g., work overload, emotional and physical demands). Consistent with studies on subjective well-being (e.g., Diener et al., 1999), the way in which teachers perceive working conditions and school relationships affects well-being at school (Kinman et al., 2011).

## Purpose of the Current Study

Based on the above described theoretical analysis, the main objective of this study is to assess the school's contribution to the teachers' well-being. Therefore, we concentrated on professional and organizational factors, which could have an influence on job and professional satisfaction and in turn on teachers' well-being in the work environment. Our study addresses the following exploratory research questions:

Q1: To what degree does the teaching working environment, management practices and policies and transformational leadership explain differences in teachers' well-being? Do these factors have varying influences on the two response variables used to tap into well-being: namely *Satisfaction with the current job environment* and *Satisfaction with the teaching profession*?
Q2: To what degree do teachers' personal and professional characteristics influence observed differences in teachers' well-being?
Q3: Which policies and practices can school principals use to support teachers' well-being?
Q4: Which policies and practices can school colleagues use to boost teachers' well-being?

## Method

### Sample and Variables

In order to investigate the influence of the above mentioned factors (teaching working environment, management practices and policies and transformational leadership) on teachers' well-being, controlling for heterogeneity, we used data collected in the large scale assessment survey named Program for International Student Assessment (PISA) carried out by the Organization for Economic Co-operation and Development (OECD) in 2015. The PISA is carried out every 3 years (since 2000) to yield comparisons of students' achievement in reading, mathematics, and science among participating countries. In 2015, for the first time, in 19 countries, the PISA surveyed teachers to inquire about different aspect of their working experience.

In this study we considered data collected in Italy where a sample of 9,738 teachers were selected. As stated in the OECD-PISA technical reports (OECD, 2019) in Italy the proportion of non-responding teachers was one of the highest, with 28.1% of sampled teachers not answering the survey^1^.

Therefore, we had a total of 7,005 completed questionnaires, and we retained for the analysis only those teachers that identified having a formal education of at least of ISCED (*International Standard Classification of Education*) Level 5. So, we considered only teachers with a tertiary degree. This is because we wanted to analyze exclusively the working context of Italian high school teachers with a similar training background. In the end, a total of 6,491 teachers were considered herein.

Datasets made available by OECD include several information, the variables considered in the analysis are described below and classified according to their characteristics. Detailed information on the survey design and variables can be found in documents and reports published at www.oecd.org/pisa/data/. Some descriptive statistics about the selected variables are reported in Table 1.

**Table 1:** Descriptive statistics.

**SEX**		
Female	4,368	67.50
Male	2,103	32.50
Missing (# obs.)	20	
**AGE**		
*M*	49.27	
*SD*	8.97	
Missing (# obs.)	16	
**TCWHAT (Subject taught in school)**		
Humanities	1,813	41.96
Maths-Science	1,976	45.73
Other	532	12.31
Missing (# obs.)	2,170	
**EMPLSTATUS (Employment status as a teacher)**		
Full time > 90%	3,507	82.56
Part time 71-90%	326	7.67
Part time 50-70%	299	7.04
Part time <50%	116	2.73
Missing (# obs.)	2,243	
**WKEXPSCH (Year working as a teacher at this school)**		
*M*	10.13	
*SD*	8.57	
Missing (# obs.)	118	
**WANNABETEACH (After completing ISCED 3-level, was your goal to pursue a career in the teaching profession?)**		
Yes	3,787	58.46
No	2,691	41.54
Missing (# obs.)	13	
**PROPDT20 (Participated in professional development in the last 12 months)**		
Yes	6,278	97.44
No	165	2.56
Missing (# obs.)	48	
**REQPROFDEV (Required to take part in professional development activities)**		
Yes	3,900	60.46
No	2,551	39.54
Missing (# obs.)	40	
**SATJOB (Satisfaction with the current job environment)**		
*M*	−0.19	
*SD*	0.94	
Missing (# obs.)	76	
**SATTEACH (Satisfaction with teaching profession)**		
*M*	0.00	
*SD*	0.93	
Missing (# obs.)	41	
**TCEDUSHORT (Educational material shortage teachers view)**		
*M*	0.25	
*SD*	1.07	
Missing (# obs.)	129	
**TCSTAFFSHORT (Staff shortage teacher view)**		
*M*	−0.08	
*SD*	0.92	
Missing (# obs.)	196	
**TCLEAD (Transformational leadership teacher view)**		
*M*	−0.19	
*SD*	0.93	
Missing (# obs.)	2,406	
**EXCHT (Exchange and co-ordination for teaching)**		
*M*	−0.18	
*SD*	0.80	
Missing (# obs.)	2,386	

### Response Variables

The two response variables have been scaled using teacher answers to different statements related to the two questions “*We would like to know how you generally feel about your job. How strongly do you agree or disagree with the following statements?*” and “*We would like to know how you generally feel about your profession. How strongly do you agree or disagree with the following statements.”* Answers have been collected using a four-point Likert scale that ranges from “strongly agree,” “agree,” “disagree” to “strongly disagree.” The derived variables were scaled by OECD using an item response theory (IRT) scaling procedure.

SATJOB. *Satisfaction with the current job environment*. Higher values of the variable point at a higher job satisfaction about the job environment. The statements considered were:

- *I enjoy working at this school*;
- *I would recommend my school as a good place to work*;
- *I am satisfied with my performance in this school*;
- *All in all, I am satisfied with my job*.

SATTEACH. *Satisfaction with teaching profession*. Higher values of the variable point at a higher satisfaction about teaching profession. The statement considered were:

- *The advantages of being a teacher clearly outweigh the disadvantages*;
- *If I could decide again, I would still choose to work as a teacher*;
- *I regret that I decided to become a teacher*;
- *I wonder whether it would have been better to choose another profession*.

The polarity of negative items has been reversed.

### Manifest Variables

PROPDT20. *Proportion of professional development*. Teachers were asked to respond to the following question: “*During the last 12 months, did you participate in any of the following activities?”* Activities included were participation in a “*qualification programme*,” a “*network of teachers focusing on professional development*,” “*individual or collaborative research on a topic of interest*,” “*mentoring and/or peer observation and coaching*,” “*reading professional literature”* and “*engaging in informal dialogue with colleagues*.” The derived binary variable PROPDT20 indicates whether a teacher took part in any of these activities in the past 12 months.

REQPROFDEV. *Required to take part in professional development activities*. Teachers were asked to respond to the Yes/No question: “*Are you required to take part in professional development activities?*.”

TCEDUSHORT. *Educational material shortage*. Teachers were asked to provide information if their school's capacity to provide instruction was hindered due to lack of educational resources (such as textbooks, libraries, laboratory materials, etc.). The four-point Likert-type scales ranged from “not at all,” “very little,” to “to some extent,” and “a lot.” Higher values indicate a higher educational material shortage.

TCSTAFFSHORT. *Educational staff shortage*. Teachers were asked whether their school's capability to operate is hampered by a lack of educational staff (such as textbooks, libraries, laboratory materials, etc.). The four-point Likert scales ranged from “not at all,” “very little,” to “to some extent,” and “a lot.” Higher values indicate a higher educational staff shortage.

TCLEAD. *Transformational leadership*. The index has been derived with an IRT scaling procedure to collect information about teachers' view on school leadership. Higher values point at a higher transformational leadership in the teachers' perception. The question considered was “*To what extent do you disagree or agree with the following statements regarding your school?”*; statements related to this question were (teachers provided their answers in a four-point Likert-type scale ranged from “strongly agree,” “agree,” “disagree” to “strongly disagree”):

- *The principal tries to achieve consensus with all staff when defining priorities and goals in school*;
- *The principal is aware of my needs*;
- *The principal inspires new ideas for my professional learning*;
- The principal treats teaching staff as professionals;
- *The principal ensures our involvement in decision making*.

EXCHT. *Exchange and co-ordination for teaching*. A question addressed to evaluate teaching-related co-operation using statements like “teaching jointly” or “exchanging teaching materials” has been considered to scale the indicator (via a IRT model). Teachers were asked to rate these activities with the following answering categories “never,” “once a year or less,” “2–4 times a year,” “5–10 times a year,” “1–3 times a month,” and “once a week or more.”

WANNABETEACH. *After completing ISCED 3-level, was your goal to pursue a career in the teaching profession?* Yes/No answers to this question have been considered.

### Control Variables

The following control variables were considered for each respondent: gender (SEX), age (AGE), years working as a teacher at her/his school (WKEXPSCH), and the employment status as a teacher (EMPLSTATUS: Full-time >90%, Part-time 71–90%, Part-time 50–70%, Part-time <50%). Moreover, the subject taught at school has been recorded (TCWHAT: Humanities, Math-Sciences, Other); note that with reference to the latter two variables we recorded a high number of missing observations, thus the variable EMPLSTATUS has not been used in the modeling approach as associated with the years of working experience and with age.

### Data Analysis Plan

In order to explore the relationship between teachers' well-being and the teachers' working environment, considering the heterogeneity between disciplines and teachers' characteristics, a bivariate regression model has been adopted. Specifically, the use of a bivariate approach allows us (Leckie and Charlton, 2013; Sulis and Porcu, 2015; Grilli et al., 2016; Leckie, 2018) to consider the effect of teacher working environment and management practices and policies on both outcome measures selected to monitor teachers' well-being: namely *Satisfaction with the current job environment* (SATJOB) and *Satisfaction with teaching profession* (SATTEACH). Both variables are treated as response variables in a regression framework with the aim to shed light on which management practices and policies may have the greatest impact on improving teachers' well-being. The use of a bivariate approach allows us to assess the level of association between the two outcome measures adjusting for the heterogeneity in teachers' characteristics (e.g., disciplines, teacher professional background and professional choices and more generally individual differences, etc.). Moreover, the explanatory model is then combined in a multilevel framework in order to assess (i) if there is a “school effect” in the heterogeneity of the results (Leckie and Goldstein, 2009), (ii) if this effect can be explained by management policies and practices in terms of leadership, poor vs. rich environment and cooperation, and (iii) the impact of these factors at different levels of the data structure (i.e., individual teacher perception *vs*. perception of teachers in the same school).

Specifically, we fit a multilevel bivariate regression model which considers at level-1 teachers and at level-2 schools and treats the two response variables as two related dimensions of a single underlying latent variable “teacher well-being.” Namely, the satisfaction of teacher *i* (*I* = 1,…, *N*) belonging to school *j* (*j* = 1,…, *J*) with respect the dimension $y_{ij}^{d}$ is specified as function of the vector of respondent (**x**) and school (**z**) covariates. Moreover, two bivariate normally distributed residual random terms $\theta_{j}^{d}\sim N\left(0,\;\Theta \right)$ and $\varepsilon_{ij\;}^{d}\;\sim N\left(0,\Sigma \right)\;$ are considered at school (Level-2) and teacher (Level-1) level to take into account for differences between schools and between teachers within the same school

(1) $$\begin{array}{l} y_{ij}^{d}=\alpha^{d}+x_{ij}^{\prime d}\beta^{d}+z_{j}^{\prime d}\gamma^{d}+\theta_{j}^{d}+\varepsilon_{ij}^{d}. \end{array}$$

**Figure 1:**
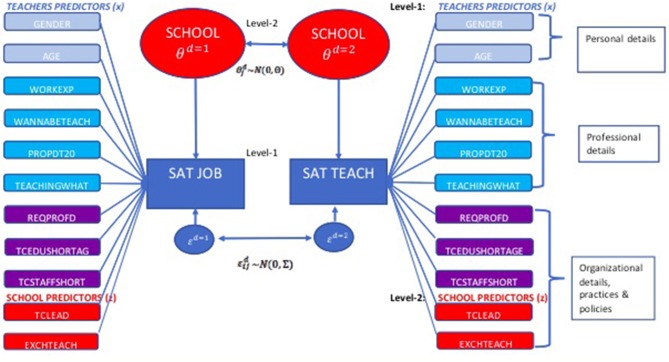
Multilevel bivariate model with teachers (Level-1 units) and schools' (Level-2 units) predictors.

As a result, the residual variability in the observed outcomes is split in the between-school and within-school components. The random term $\theta_{j}^{d}$, which is shared by teachers belonging to the same school, captures the school “net” effect on the two measures of teachers' well-being (Goldstein and Spiegelhalter, 1996; Goldstein, 2011). The bivariate multilevel model relationship described in Equation 1 is displayed in Figure 1. The random term $\varepsilon_{ij}^{d}$ captures the residual variability between the responses of teachers belonging to the same school. The model has been estimated with STATA using the runMLwiN routine implemented by Leckie and Charlton (2013), by specifying as the estimation method the Iterative Generalized Least Squares. The bivariate approach allows us to assess the degree of correlation between the two dimensions of teachers' well-being: teachers' satisfaction with respect to their current job and their profession, to assess the effect of covariates on both components and to control the effect of the confounding factors (control variables) on both dimension of well-being.

## Results

We adopted a model building strategy to assess the determinants of teachers' well-being extending a bivariate null model (M0) in several directions. Namely, the bivariate model has been generalized considering the clustering of teachers in schools and the heterogeneity between teachers and schools in terms of their characteristics. Predictors have been clustered in three groups: (i) teacher personal and professional details (SEX, AGE, WKEXPSCH, TCWHAT, PROPDT), including the teacher's self-stated motivation toward the profession (WANNABETEACH); (ii) teacher's perception of the school working environment in terms of resources, organizational details, practices and polices (REQPROFDEV, TCEDUSHORTAGE, TCSTAFFSHORTAGE, TCLEAD, EXCHTEACH); (iii) compositional variables at school level obtained as the average at the school level of predictors mentioned in point (ii).

To manage the high rate of missing values in some variables, the information provided by variables TLEAD and EXCHTEACH has been considered only at the school level, taking for both variables the average value over teachers belonging to the same school. Moreover, missing values observed for the variable TCWAHT have been treated as a further category of the variable, defining four possible response categories (not declared, mathematics, language, others). We have taken this decision for two main reasons: (1) to inspect if there were differences in the two dimensions of perceived well-being related to belonging to one of the two clusters of teachers (math and language) with respect to other clusters and (2) to assess if there were any differences between observations with missing values and the others. The decision was also supported by the fact that language and maths are the two main disciplines which provide students the competences investigated in the main large scale assessment surveys carried out at both national and international level. So we want to inspect if the was a kind of “main disciplines” effect in teachers' perception of the two dimensions of well-being. Also some studies have suggested that teaching subjects could have an effect on job satisfaction and competence that in turn affect teacher well-being (see for example Pillay et al., 2005; Kunter et al., 2011; Ilgan et al., 2015). The variable AGE has been split into three intervals: <36, 36–50, >50. Although relevant literature for the Italian context, uses a different way to categorize age (namely, <39, 40–54 and >55) (see, for example James et al., 2011; Avanzi et al., 2012), this clustering of the variable has allowed us to maximize differences across observations in the effect of age on the two dimensions of perceived well-being. We did not consider the variable as continuous and we applied this categorization in order to better differentiate the effect of the predictors on the response variable. Finally, given the rate of missingness in almost all variables, 5,973 complete observations (92%) have been retained for the final analysis. Overall, five competing models (Models 0–4 [M0-M4]) were estimated and results compared in terms of goodness of fit statistics (Bayesian Information Criterion [BIC] and Akaike information Criterion [AIC]), which are summarized in Table 2. The model comparisons included comparing the variance component model (M1) to the null model (M0) to assess the relevance of the clustering of students in schools. We also compared the multilevel model with teacher's predictors (M2) with the variance component model (M1) to assess the relevance of teachers' characteristics defined at point (ii) in terms of personal and professional details and perception of the school working environment in terms of resources, organizational details, practices and policies on the two outcomes. Furthermore, the multilevel model with compositional variables at school level (M3), has been estimated to assess the effect of school compositional variables in terms of organizational aspects, practices and policies on teachers' well-being. The comparison between M3 and M2 shows that the introduction of compositional variables at the school level (M3) to determine if a greater improvement of goodness of fit statistics with respect to the introduction of predictors at the teacher level (M2) exists. This finding suggests that school management in terms of transformational leadership (the only significant predictor) has the greatest impact on teacher well-being. Finally, M4 which assesses the effect of relevant predictors at teacher and school level considering both sets of covariates, combines findings from M2 and M3. Comparisons between bivariate models in terms of goodness of fit statistics in Table 2 show that M4 is the model which the data better than all other models. Thus, it is championed for analyzing differences in well-being between teachers and to shed some light on its determinants.

**Table 2:** Model comparisons: null bivariate vs. bivariate multilevel linear models.

**Variable**	***M0***		***M1***		***M2***		***M3***		***M4***	
	**(I) TEACHER PERSONAL PROFESSIONAL DETAILS & MOTIVATION**									
	***SATJ***	***SATT***	***SATJ***	***SATT***	***SATJ***	***SATT***	***SATJ***	***SATT***	***SATJ***	***SATT***
**CONS**			−0.186^***^	0.00144	−0.281^***^	−0.260^***^	−0.0681^**^	0.108^***^	−0.207^**^	−0.188^**^
			(0.0178)	(0.0169)	(0.0929)	(0.0903)	(0.0282)	(0.0306)	(0.0910)	(0.0911)
SEX (M)					0.00456	−0.00439			−0.0105	−0.0173
					(0.0261)	(0.0255)			(0.0252)	(0.0253)
AGE (35-50)					−0.224^***^	−0.191^***^			−0.161^***^	−0.148^***^
					(0.0466)	(0.0455)			(0.0450)	(0.0451)
AGE (>50)					−0.336^***^	−0.467^***^			−0.243^***^	−0.397^***^
					(0.0497)	(0.0484)			(0.0480)	(0.0482)
**TCWHAT (MISSING)**										
(HUM)					0.0155	0.0280			−0.00625	0.0166
					(0.0311)	(0.0303)			(0.0300)	(0.0300)
(SCIENCE)					0.0384	0.0834^***^			0.0433	0.0869^***^
					(0.0299)	(0.0292)			(0.0289)	(0.0289)
(OTHER)					0.116^**^	0.0705			0.126^***^	0.0733
					(0.0467)	(0.0457)			(0.0450)	(0.0451)
WKEXPSCH					0.00204	0.00518^***^			0.00152	0.00447^***^
					(0.00162)	(0.00158)			(0.00156)	(0.00157)
WANNABET (YES)					0.208^***^	0.424^***^			0.169^***^	0.407^***^
					(0.0248)	(0.0242)			(0.0240)	(0.0240)
PROPDT20					0.191^**^	0.242^***^			0.189^**^	0.234^***^
					(0.0780)	(0.0761)			(0.0753)	(0.0753)
**TEACHER**	**(II) ORGANIZATIONAL DETAILS, PRACTICES & POLICIES**										
REQPROFD (NO)							−0.122^***^	−0.104^***^	−0.113^***^	−0.0846^***^
							(0.0241)	(0.0248)	(0.0240)	(0.0240)
TCEDUSHORT							−0.132^***^	−0.111^***^	−0.126^***^	−0.0976^***^
							(0.0125)	(0.0128)	(0.0120)	(0.0121)
TCSTAFFSHORT							−0.162^***^	−0.0569^***^	−0.160^***^	−0.0484^***^
							(0.0139)	(0.0143)	(0.0137)	(0.0137)
**SCHOOL**	**(III) ORGANIZATIONAL DETAILS, PRACTICES & POLICIES**										
TCEDUSHORT							−0.0172	−0.00843		
							(0.0442)	(0.0478)		
TCLEAD							0.235^***^	0.110*	0.233^***^	0.0951*
							(0.0534)	(0.0580)	(0.0490)	(0.0494)
TCSTAFFSHORT							−0.0303	−0.0625		
							(0.0798)	(0.0868)		
EXCHTEACH							0.00294	0.132	−0.0129	0.117
							(0.0821)	(0.0892)	(0.0820)	(0.0827)
		**Level-1**	**Level-2**	**Level-1**	**Level-2**	**Level-1**	**Level-2**	**Level-1**	**Level-2**	**Level-1**
var(cons_d1)		0.877^***^	0.0204^***^	0.856^***^	0.0189^***^	0.837^***^	0.00673^**^	0.794^***^	0.00745^**^	0.781^***^
		(0.0161)	(0.00495)	(0.0158)	(0.00471)	(0.0155)	(0.00295)	(0.0147)	(0.00302)	(0.0145)
cov(cons_d1/cons_d2)		0.431^***^	0.00782^**^	0.422^***^	0.00497	0.390^***^	−0.00119	0.385^***^	−0.000927	0.360^***^
		(0.0127)	(0.00364)	(0.0125)	(0.00311)	(0.0119)	(0.00236)	(0.0118)	(0.00222)	(0.0113)
var(cons_d2)		0.881^***^	0.0164^***^	0.865^***^	0.0107^***^	0.803^***^	0.0103^***^	0.840^***^	0.00781^**^	0.785^***^
		(0.0161)	(0.00443)	(0.0160)	(0.00351)	(0.0148)	(0.00355)	(0.0155)	(0.00307)	(0.0145)
Observations	5,973	5,974	5,973	5,973	5,973	5,973	5,973	5,973	5,973	5,973
Number of groups			126	126	126	126	126	126	126	126
Deviance	30,681	30,681	30,592	30,592	30,113	30,113	30,065	30,065	29,637	29,637
AIC	30,691	30,691	30,608	30,608	30,165	30,165	30,109	30,109	**29,709**	**29,709**
BIC	30,681	30,681	30,592	30,592	30,113	30,113	30,065	30,065	**29,637**	**29,637**
Log likelihood	−15,340	−15,340	−15,296	−15,296	−15,057	−15,057	−15,032	−15,032	−14,818	−14,818
*k*	5	5	8	8	26	26	22	22	36	36
numlevels	1	1	2	2	2	2	2	2	2	2

*Standard errors in parentheses*.

*** *p < 0.01*.

** p < 0.05. *p < 0.10.

### Key Findings

Results depicted in Table 2 for M4 display a medium to low level of correlation between the two outcome variables (0.48) suggesting that they play a key role in assessing teaching well-being. Also older teachers have on average a general level of satisfaction lower than younger teachers. Particularly strong is the effect of belonging to the highest age group on satisfaction toward the teaching profession (on average the oldest age group of teachers are 0.4 less satisfied than the youngest group of teachers). Teachers who had the ambition to pursue a career in the teaching profession are on average more satisfied with respect to both dimensions of well-being as it has been operationalized, with a greater impact on the satisfaction toward the teaching profession. Moreover, the number of years of working experience in the school where they currently work seems to have a positive influence only on satisfaction toward the profession. With respect to the disciplines, results suggest that science teachers show on average a greater level of satisfaction with respect to their profession than the other groups, while satisfaction with respect to the working environment seems to be higher for teachers in minor subjects. Teachers who invested in professional development in the last 12 months are on average both satisfied with respect to both dimensions. The main finding concerning organizational aspects, management policies and practices provided by M4 (see Table 2) suggest that the teachers' perception of a poor working environment, in terms of shortage of resources and teaching staff, has a negative impact on both dimensions of teachers' well-being, but a stronger impact on satisfaction toward the current job environment (TCEDUSHORTAGE = −0.13 and TCSTAFFSHORTAGE = −0.16). Also, the shortage of teaching resources has the greatest impact on satisfaction with the current job environment, whereas the shortage of educational resources has a greater influence on the satisfaction with teaching profession. The school policy to require teachers to take part in professional development activities (REQPROFDEV) provides an expected increase of +0.11 of the score of the teacher's satisfaction with the current job environment and positively affect the satisfaction also with teaching profession (+0.08). Looking at the role of the school principals, it arises that the transformational leadership teachers view is a management practice that has a relevant impact on teachers' well-being. Namely if we compare the value of two schools with two extreme positions (i.e., minimum = −1.03 vs. maximum value = 0.64) with respect the transformational leadership factor it arises that this practice explains about 0.4 standard deviation in the difference in teachers' satisfaction with respect their job, weaker is the effect on satisfaction with respect to their profession. Teachers' perception of a cooperative environment, which promotes exchange and co-ordination for teaching, does not show a relevant effect. This could be explained by the association of this aspect with other practices. The comparison between M0 and M4 also shows that the introduction of teachers and school covariates among the predictors explain almost all the between school variability and produces a decrease of about 11% of the within school variability. To sum up, after controlling for teacher heterogeneity in age, disciplines, working experience and career choices (i.e., WANNABETEACH and PROPDT), it arises that the perception that teachers' have of school resources together with the implementation of policies which support professional development can have a great impact on both aspects of teaching well-being. Moreover, as expected, teachers' well-being benefits of policies and practices that school can implement in terms of supporting transformational leadership, whereas seems not been related to the perception teachers' have of the coordination for teaching. It is worth to highlight that almost all variability in the expected outcomes is due to unexplained individual differences and that the residual variability at school level is very low compared with the one observed at teacher level. However, the residual variability at school level is significant in model M1-M4. Moreover, the value added of a multilevel approach instead a simple bivariate approach is that it allows us to deal with covariates at school-level and to assess their impact on individual well-being.

## Discussion

The focus of this study was to investigate factors influencing and enhancing well-being among teachers in Italy. The modeling approach here adopted allows us to shed light on determinants of the differences in teachers' well-being and to differentiate on their impact on the two outcome measures (Research Question –RQ– 1). Specifically results provide evidence that a teacher's professional background together with their experience, organizational aspects and school practices and policies have a relevant impact on both outcome measures as they relate to well-being. The impact of teacher personal and professional details is stronger on satisfaction toward the teaching profession whilst, as expected, the effect of organizational aspects, practices and policies is stronger on the satisfaction toward the current job.

Concerning personal and professional details that mainly affect observed differences in teachers' well-being (RQ 2) results suggest that younger teachers, with a greater working experience, who pursued a specific training to become a teacher and invested in professional development experienced on average higher levels of well-being than older teachers. Question regarding number of years of teaching experience is however controversial, in fact some studies reported that longer teaching experience is associated with higher levels of job satisfaction (Taylor and Tashakkori, 1995), on the contrary others reported that teachers who have taught longer are less satisfied (Xin and MacMillan, 1999), thus continuing the debate and encouraging further research.

Moreover, findings highlight that policies and practices that school principals can support to improve teachers' well-being (RQ 3) are those which support a transformational leadership teachers' view involve the investment in professional development and the availability of educational resources for teachers. As other studies have shown, the support of the principal, in terms of encouragement and involvement in participatory decision-making processes has positive repercussions on the well-being of teachers (Singh and Billingsley, 1996; Howard and Johnson, 2004). Finally, no evidence was provided in the data about the effectiveness of policies and practices that school colleagues can implement to support teachers' well-being in terms of exchange and coordination for teaching (RQ 4).

The identification of factors with a positive effect might enable schools to undertake actions to enhance teachers' well-being. The school working environment factor indeed plays an important role in teachers' well-being, and has to do with the school availability of resources, and/or local practices and policies. Results like this one provide evidence that there are indeed factors influencing teachers' well-being which can be optimized at the school level. These findings are in accordance with the theoretical framework described by Bakker and Demerouti (2014).

Findings also support the idea that principals play a pivotal role in steering the direction of their school which requires guiding the day-to-day business of the school including matters associated with both students and teachers. Furthermore, the growing autonomy of Italian schools compared to the ministerial and regional authorities is creating confused dynamics with respect to organizing school activities. This element became particularly evident with the latest educational reform (Law 107/2015), which increased the level of autonomy of the principal with respect to the evaluation and management processes inside the school. From a practical point of view, our results highlight the importance of the “school effect,” in terms of teachers' perception of having both material and staff resources at their disposal in work context. At the same time, the possibility of continuous professional development, and involvement in the management processes in school environment result in influencing teachers' satisfaction and well-being. It is therefore essential that school leaders try to create a stimulating and supportive environment in order to promote the professional growth of their teachers.

## Limitation and Future Research

This is an exploratory study which provides some initial evidence on factors affecting well-being among Italian teachers. However, the present study has several limitations. First, the high rate of missing values in some variables forced authors choices of variables to consider in the analysis and aggregation of the variables at school level instead that at teacher level. Second, the availability of data related to a single wave of the PISA survey and to a single large scale assessment survey did not allow us to generalize the results over time. Since we were interested in the assessment of the effect of organizational aspects, practices and policies in affecting teachers' well-being, we focused on influences of the organizational context controlling for teachers' heterogeneity with respect to professional and personal details using cross-national data. Moreover, information related to the investment in professional development actions has been summarized in general terms in the PROPDT20 variable, using responses related to different practices. A deeper analysis of the effect of different actions described in the items on well-being dimensions would be recommended in order to address teachers and principals in supporting policies which can provide the greatest value added. Considering the fact that our sample consisted of teachers in secondary schools, it would be interesting to test the consistency of the results in other educational frameworks and other countries. For this reason, we believe that futures researchers should include additional future waves of the PISA survey and should consider teachers of different school levels and countries.

## Data Availability

Publicly available datasets were analyzed in this study. This data can be found here: www.oecd.org/pisa/data/.

## Ethics Statement

All participants gave their written informed consent before the administration of the questionnaire, in accordance with the Declaration of Helsinki. The study was carried out in accordance the rules of AIP (Associazione Italiana di Psicologia—Italian Association of Psychology), according to which there was no need for previous ethics approval, since it would not deal with animals or vulnerable groups, or would involve risk for the well-being of participants, or use biomedical devices, or invasive investigation tools. Our study did not need ethics approval, according to our national regulations as well as to the Ethics Committee of the University of Cagliari.

## Author Contributions

BB developed the research project, with the contribution of IS, MP, and MT. MP prepared the data set. IS carried out the data analysis. BB reviewed the literature. MT reviewed and edited the paper.

### Conflict of Interest Statement

The authors declare that the research was conducted in the absence of any commercial or financial relationships that could be construed as a potential conflict of interest. The handling editor declared a past collaboration with one of the authors BB.

## Footnotes

^1^Countries with the highest percentage of non-respondents were Australia, Brazil, Germany and Italy, each with about 30% of the total. For these countries, results should be interpreted with caution as they might be affected by possible non-response bias (OECD, 2019).
